# Investigating the relationship between hypoxia, hypoxia-inducible factor 1, and the optical redox ratio in response to radiation therapy

**DOI:** 10.1117/1.BIOS.1.1.015003

**Published:** 2024-05-28

**Authors:** Jesse D. Ivers, Nagavenkatasai Puvvada, Charles M. Quick, Narasimhan Rajaram

**Affiliations:** a University of Arkansas, Department of Biomedical Engineering, Fayetteville, Arkansas, United States; b University of Arkansas for Medical Sciences, Department of Pathology, Little Rock, Arkansas, United States

**Keywords:** multiphoton microscopy, radiation resistance, cancer, histogram analysis, multivariate, tumor microenvironment

## Abstract

**Significance:**

Radiation resistance is a major contributor to cancer treatment failure and is likely driven by multiple pathways. Multivariate visualization that preserves the spatial co-localization of factors could aid in understanding mechanisms of resistance and identifying biomarkers of response.

**Aim:**

We aim to investigate the spatial and temporal relationship between hypoxia, hypoxia-inducible factor 1 (HIF-1α), and metabolism in response to radiation therapy in two cell lines of known radiation resistance and sensitivity.

**Approach:**

Two-photon excited fluorescence and fluorescence lifetime imaging microscopy were used to quantify the optical redox ratio (ORR) and NAD(P)H fluorescent lifetime and bound fraction in frozen tumor sections and co-registered with immunohistochemical stain-based imaging of hypoxic fraction and HIF-1α.

**Results:**

Histogram analysis of hypoxia, HIF-1α, and ORR revealed an increase in the ORR in regions of low hypoxia and high HIF-1α, indicating that the stabilization of HIF-1α is likely due to an increase in reactive oxygen species following radiation therapy. In addition, the bound NAD(P)H fraction was higher in regions with a low ORR in resistant tumors following radiation, suggesting an increase in fatty acid synthesis.

**Conclusions:**

A multivariate histogram approach can reveal hidden trends not observed in bulk analysis of tumor images and may be useful in understanding biomarkers and mechanisms of radiation resistance.

Statement of Discovery This work uses multivariate histogram analysis of two-photon excited autofluorescence images acquired from excised tumor sections to investigate the relationship between tumor hypoxia and metabolism in radiation resistance.

## 1 Introduction

The majority of patients with head and neck squamous cell carcinoma (HNSCC) are treated with radiation therapy (in addition to surgery and/or chemotherapy and immunotherapy). Unfortunately, nearly 30% of patients will experience loco-regional recurrence following treatment.^1^^–^^4^ Oxygen is a key ingredient in radiation’s success as radiation creates free radicals in DNA or water molecules that react with the available oxygen to cause ionizing damage to the DNA.^5^^,^^6^ Tumor hypoxia, on the other hand, leads to treatment failure due to the lack of this key vehicle.^7^^–^^9^ Hypoxia leads to the stabilization of hypoxia-inducible factor 1 alpha (HIF-1α), a master regulator of oxygen homeostasis.^10^ In cancer cells, HIF-1α stabilization promotes angiogenesis and activates several downstream glycolytic genes that promote solid tumor growth.^11^^–^^14^ The level and intensity of HIF-1α expression is inversely correlated with disease-free survival in cancers of the oropharynx treated with radiation therapy.^15^ However, studies have shown that factors other than hypoxia, such as radiation-induced reoxygenation within the tumor, can lead to an increase in reactive oxygen species, leading to the activation of HIF-1α^16^^,^^17^ and a subsequent increase in glucose uptake.^18^ Increased glucose catabolism can promote radiation resistance through utilization within the pentose phosphate shunt to maintain the NADPH-glutathione buffer and hence scavenge radiation-induced ROS. Thus, although hypoxic tumors respond poorly to radiation therapy, well-oxygenated tumors can also escape the effects of radiation by leveraging alternative metabolic pathways.

The determination of the relationship between oxygenation and metabolism has typically involved measurements of glucose uptake and lactate production in cells *in vitro* in the presence and absence of oxygen.^19^ However, these studies do not account for tumor heterogeneity; it is well established that tumor hypoxia varies both in space and time across a tumor,^20^ leading to likely similar perturbations in HIF-1α and hence metabolic pathways. Therefore, there is a need to understand how the relationship between hypoxia, HIF-1α, and metabolism affects the response to radiation therapy in tumors.

Two photon microscopy can provide label-free, high-resolution, and quantitative readouts of cell metabolism. Endogenous fluorescence intensities of the metabolic cofactors reduced nicotinamide adenine dinucleotide, and the spectrally indistinguishable NAD(P)H as well as oxidized flavin adenine dinucleotide (FAD)^21^^,^^22^ can be used to quantify the optical redox ratio (ORR), defined here as $\mathrm{ORR}=\frac{I_{\mathrm{FAD}}}{I_{\mathrm{NAD}(P)H}+I_{\mathrm{FAD}}}$. This metric has become a well-established means of assessing cellular redox state and distinguishing between sub-populations of cells.^23^^–^^26^ Fluorescence lifetime imaging microscopy (FLIM) provides a measure of the average time spent by a fluorophore in the excited state before decaying to its ground state. Protein-bound NAD(P)H has a relatively long lifetime, from 1.9 to 5.7 ns, whereas free NAD(P)H has a shorter lifetime, around 0.4 ns.^27^^–^^29^ Protein-bound NAD(P)H lifetime can be more sensitive than the ORR or mean lifetime in determining metabolic changes associated with the diversion of glucose away from its traditional pathway to the mitochondria.^30^

Previous studies in our lab have investigated the relationship between HIF-1α and the ORR *in vitro* in response to radiation therapy and found that an increase in HIF-1α was accompanied by a decrease in the ORR in radiation-resistant cancer cells treated with radiation, suggesting an increase in glucose catabolism.^31^ Knockdown of HIF-1α led to an increase in the ORR that was accompanied by a decrease in glucose uptake, a decrease in reduced glutathione, and an increase in ROS in radiation-resistant cells, demonstrating that the radiation-resistant cells were shunting glucose through the pentose phosphate pathway to generate NADPH that could maintain the pool of reduced glutathione to scavenge free radicals.^32^ Immunohistochemical assessment of HNSCC tumors excised from mice following radiation therapy showed a significantly higher accumulation of HIF-1α in treatment-resistant tumors compared with treatment-sensitive tumors, despite only minor differences in overall hypoxic fraction.^33^ To our knowledge, no study has examined the spatiotemporal dynamics of hypoxia, HIF-1α, and metabolism within tumors, especially in the context of radiation therapy.

The objective of this study is to investigate the relationship between hypoxia, HIF-1α, and the ORR in radiation-sensitive and resistant cancer cells prior to treatment and in response to treatment. We used two human HNSCC lines: UM-SCC-22B (radiation-sensitive) and UM-SCC-47 (radiation-resistant). These cell lines were grown as tumor xenografts in mice and excised either prior to therapy or 24 and 48 hr following radiation therapy. Whole-section autofluorescence imaging of NAD(P)H and FAD and immunohistochemical stain-based imaging of hypoxia and HIF-1α were performed on the same tumor sections using two-photon microscopy. In addition, we acquired intensity and lifetime maps from specific fields of view within each section. Trivariate histograms of co-registered images of hypoxic fraction, HIF-1α, and the ORR revealed relationships between the three parameters not apparent when analyzing the bulk mean of each parameter across the entire tumor section. Specifically, we found regions of low-hypoxia that had high HIF-1α expression and increased ORR, a finding at odds with the traditional observation of a reduced ORR under hypoxic conditions and high HIF-1α expression. In addition, analysis of the relationship between fluorescence lifetime and ORR revealed regions of increased bound fraction in regions with low ORRs in the resistant tumors following radiation therapy, consistent with optical metabolic changes associated with fatty acid synthesis, which has been shown to promote radiation resistance in cancer cells. Altogether, we show that multidimensional imaging and analysis can provide a deeper view into the tumor microenvironment, especially the relationship between tumor oxygenation and metabolism that is known to play key roles in radiation resistance.

## 2 Materials and Methods

### 2.1 Cell Culture

Cell culture conditions, xenograft protocol, and radiation treatment procedures have all been detailed previously.^33^^–^^35^ In summary, UM-SCC-22B (22B) (established from HPV 16- metastatic lymph node of a female patient) and UM-SCC-47 (47) (established from HPV 16+ primary tumor of lateral tongue of a male patient) were purchased from EMD Millipore and cultured in Dulbecco’s modified Eagle medium with 10% fetal bovine serum, 1% penicillin-streptomycin, 1% non-essential amino acids, and 1% L-glutamine.

### 2.2 Tumor Xenografts and Radiation Treatment

**Fig. 1:**
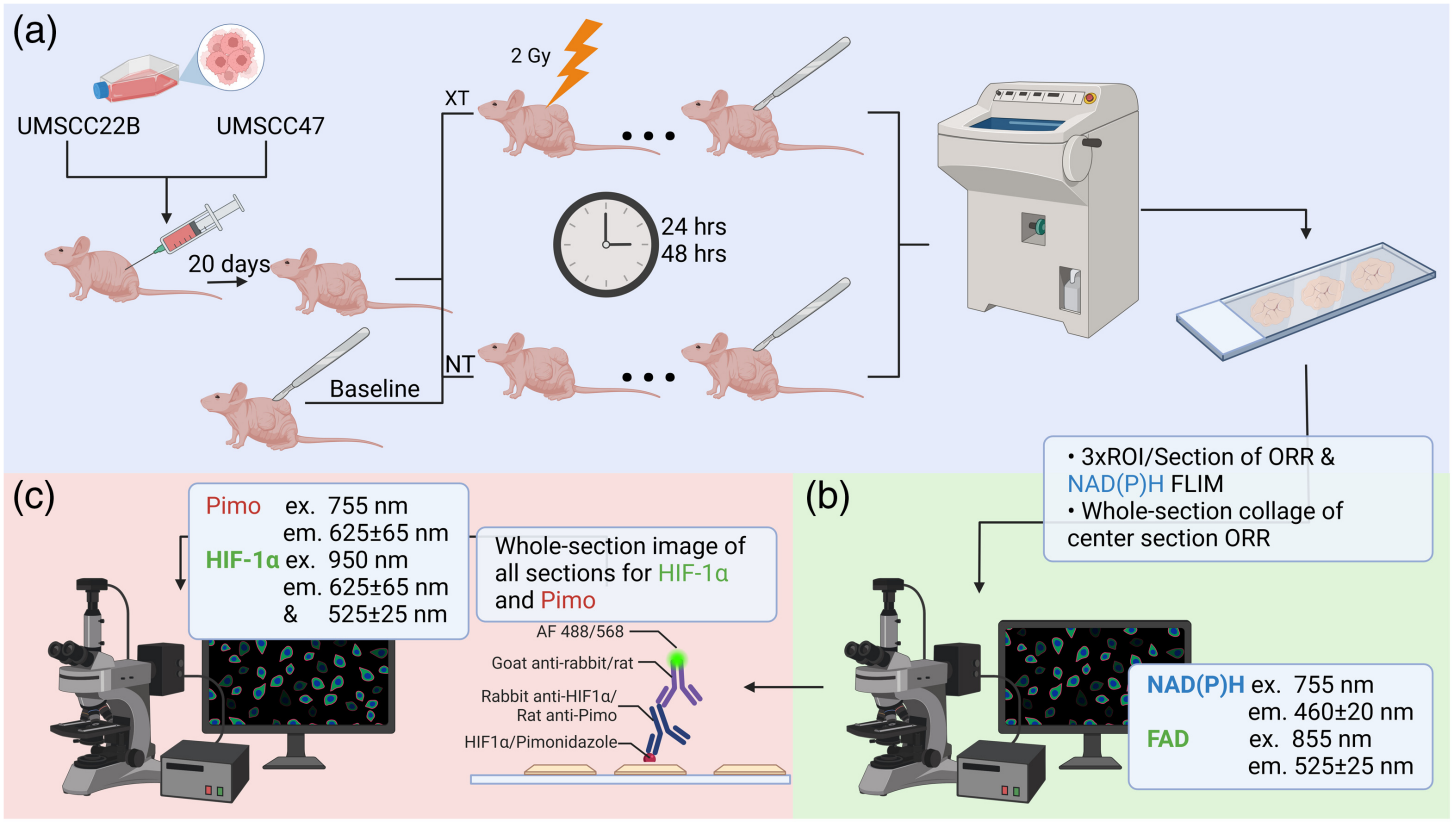
Schematic of the study design and methods. (a) Timeline of tumor implantation and group divisions. (b) Overview of the autofluorescence imaging protocol. (c) Overview of the IHC staining and imaging protocol for pimonidazole and HIF-1α. Created with BioRender.^36^

All animal studies were approved by the Institutional Animal Care & Use Committee at the University of Arkansas (protocol number: 18061). Mice were purchased from Jackson Laboratories (Bar Harbor, Maine) and housed at the Central Laboratory Animal Facility with *ad libitum* access to food and clean water and standard 12 hr light/dark cycles. After a 2 to 3 week acclimatization period, 1.5 million cells suspended in a 1:1 mixture of Matrigel (Corning, Corning, New York) and saline were injected into the right flank (for treatment groups) or both flanks (for control groups) of athymic (nu/nu) mice to form xenografts. This study included 23 mice in the UM-SCC-22B group and 18 mice in the UM-SCC-47 tumors. Mice from each tumor group were further divided into treatment (XT) or control (NT) groups (n=2 to 5 in each group). Animals in the XT group were treated with a single dose of 2 Gy of radiation using an X-Rad 320 biological cabinet (Precision X-Ray, North Branford, Connecticut) while under anesthesia using a 1.5% v/v isoflurane and oxygen mixture. The animal’s body was covered using lead blocks except for the tumor. Animals in the NT group served as controls and did not undergo radiation. XT and NT groups were subdivided into groups for euthanasia and tumor excision time relative to treatment: baseline (before treatment) and 24 and 48 hours post-treatment (hr). When tumor volume reached $200\;{\mathrm{mm}}^{3}$, (∼20 days after tumor implantation), mice underwent the assigned treatment (NT/XT), and tumors were excised at the assigned time [Fig. 1(a)].

### 2.3 Immunohistochemistry

Mice were injected (i.p.) with pimonidazole (60  mg/kg; Hypoxyprobe, Burlingame, Massachusetts) 1 hr prior to euthanasia. Pimonidazole (pimo) is a nitroimidazole that forms adducts with thiol-containing proteins in hypoxic cells and tissue.^37^ Tumors were excised, flash-frozen, and later sectioned with 50  μm thickness using a cryostat (CM 1860; Leica, Inc., Nusslock, Germany). Each slide contained three serial sections. Slides were stored at −80°C before and after imaging. Immunohistochemistry (IHC) was performed on slides after autofluorescence imaging to isolate endogenous and exogenous signals.

An indirect IHC staining procedure was used to detect hypoxia (through pimo labeling) and HIF-1α accumulation within a single tissue section. Reagent concentrations and incubation times were optimized through in-house trials to maximize the signal and minimize the non-specific background. Briefly, the slides were warmed to room temperature from −80°C, and individual sections were outlined with a hydrophobic pap pen (H4000; Vector Laboratories, Burlingame, California). Slides were fixed with 4% PFA, washed three times with PBS for 2 min, and permeabilized with 0.5% Triton-X 100. Off-target antigen binding was blocked with an in-house blocking solution of 96% PBS, 4% goat serum, and 1% sodium azide for 1 hr at room temperature. The blocking solution was aspirated, and a primary antibody solution, composed of 0.5% Rabbit anti-HIF-1α (36169; cell signaling, Danvers, Massachusetts), 2% Rat anti-pimo (Rat Mab; Hypoxyprobe, Burlington, Massachusetts) and 97.5% blocking solution, was added. Slides were incubated in the primary antibody solution for 3 h at room temperature and then washed three times in PBS for 2 min. A secondary antibody solution of 1% Goat anti-Rabbit Alexa Fluor 488 (A27034; Invitrogen, Waltham, Massachusetts), 1% Goat anti-Rat Alexa Fluor 568 (A11077; Invitrogen, Waltham, Massachusetts), and 98% blocking solution was added, and slides were incubated for 45 min at room temperature. These conjugate fluorophores were selected to optimize the signal separation into separate channels (further described in IHC imaging and signal extraction). Slides were washed three more times for 2 min in PBS, then mounted with fluoromount-G (0100-01; SouthernBiotech, Birmingham, Alabama), covered and sealed with nail polish, and allowed to dry overnight.

### 2.4 Two Photon Excited Fluorescence

All imaging (autofluorescence—intensity and lifetime—and IHC assays) was performed using two photon excited fluorescence (TPEF) with excitation using a tunable Ti:Sapphire laser (Spectra-Physics, Santa Clara, California) and emission detected in three distinct channels using GaAsP photomultiplier tubes (PMT) (H10770PB-40; Hamamatsu, Shizuoka, Japan). The center wavelength and bandpass for each emission filter (ET680sp-2p; Chroma, Bellows Falls, Vermont) were as follows: 460/40  nm (blue), 525/50  nm (green), and 625/30  nm (red). All images were captured with a 20×, 1.0 NA water-immersion objective (Olympus, Tokyo, Japan). Individual images (whether a region of interest image or a single frame of a whole-section image) were captured with a pixel dwell time of 3.8  μs at a size of 512×512  pixels (584 584×584  μm) and a 13-bit depth. Power and PMT gain were manually adjusted to maximize the SNR and recorded for normalization after imaging (see Sec. 2.5). The incident power was never allowed to exceed 65 mW. All whole-section images were captured using Prairie View’s (Bruker Corporation, Billerica, Massachusetts) Atlas Imaging application with a frame overlap of at least 15%. Specific imaging details for each assay are described in the following sections and graphically in Figs. 1(b) and 1(c).

#### 2.4.1 Autofluorescence intensity and lifetime

NAD(P)H was excited at 755 nm and emission detected in the blue channel. FAD was excited at 855 nm and emission detected in the green channel. Whole-section autofluorescence was captured from one tumor section/slide to limit the amount of time at room temperature and ensure minimal changes in autofluorescence as a result of freeze thaw^38^. Whole-section imaging required ∼30  min to acquire both NAD(P)H and FAD signals. A multi-level nested ANOVA (see Sec. 2.6) revealed no significant variations between individual region of interest (ROI) images or due to sections, confirming the signal consistency over the imaging time. During protocol optimization, IHC signals from two sections were used to validate the imaging protocol; one section was imaged exclusively for IHC signals, and the other was imaged for endogenous signals first, undergoing a single thaw-freeze cycle. Images from these samples were observed, and it was determined that there was no evident degradation of the IHC signal intensity or quality due to the prior autofluorescence imaging or limited time at room temperature. The fluorescence lifetime of NAD(P)H was imaged using time-correlated single-photon counting (SPC-150; Becker & Hickl Gmbh, Berline, Germany). FLIM acquisition was performed over a 2 min integration time with an 80 MHz laser pulse and 256 0.0390625 ns temporal bins to ensure a sufficient number of photon counts to determine lifetime components and distinguish lifetime species. Autofluorescence intensity and lifetime images were captured for at least three ROIs for each section [Fig. 1(b)].

#### 2.4.2 IHC imaging and signal extraction

**Fig. 2:**
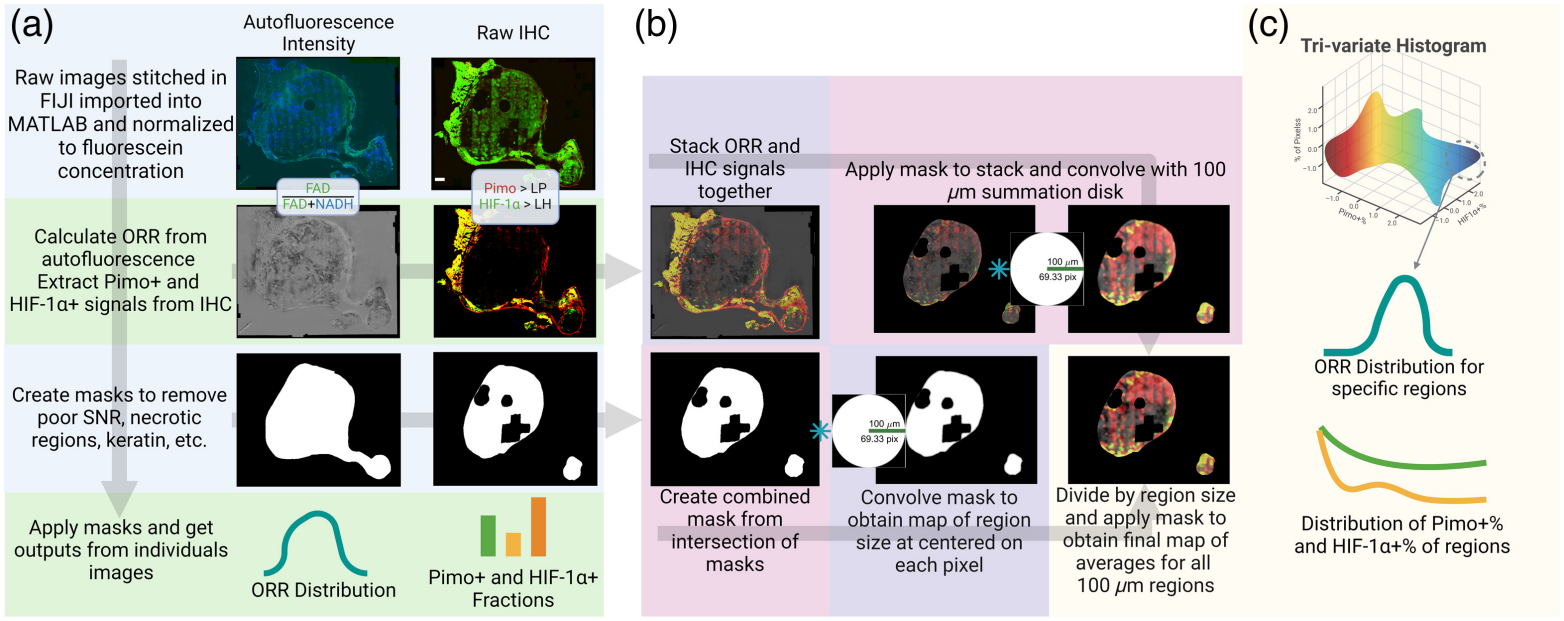
Schematic of image processing for whole-section images. (a) Univariate processing (following vertical arrows) of autofluorescence intensity and IHC signals with intermediate outputs. LP and LH are representative of the threshold cutoff values for pimo and HIF-1α stains to obtain the resultant binarized IHC signal map. Red and green pixels in the binarized IHC map represent pimo+ and HIF-1α+ staining, respectively. In regions of overlap and near overlap, the RGB representation appears yellow. (b) Multivariate processing derived from univariate maps (following horizontal arrows) with masking and regional analysis of image stack to produce (c) final multivariate outputs. Created with BioRender.^36^

Following IHC, Alexa Fluor 568 was excited at 755 nm and Alexa Fluor 488 at 950 nm. Two-photon excitation for Alexa Fluor 488 and Alexa Fluor 568 has been reported previously.^39^ The spectral overlap between the 755nm and 950 nm excitation wavelengths was selected and determined experimentally to maximize the total emission of fluorophores while minimizing any overlap from endogenous fluorophores. To confirm this, serial sections were imaged under identical parameters for autofluorescence intensity and IHC staining intensity to determine the effects (if any) of endogenous fluorophores on IHC signals. Under these conditions, autofluorescence intensity was negligible compared with the intensity from exogenous fluorophores, which were typically orders of magnitude more intense. As a result of this, any endogenous fluorescence signal present under the IHC imaging conditions was effectively removed by thresholds and binarization during processing. Whole-section IHC images were captured using Prairie View’s Atlas Imaging from three sections/slide [Fig. 1(c)]. Pimo+ and HIF-1α+ pixels were determined after fluorescein normalization using intensity thresholds that were determined by averaging multiple manually set thresholds across a random subset of images. Pimo+ pixels were isolated from the red channel of 755 nm excitation images, and HIF-1α+ pixels were isolated from the product of the red and green channels of 950 nm excitation images [Fig. 2(a)]. This product was found experimentally to minimize the background staining, maximize the true signal, and improve the signal-to-noise ratio for low HIF-1α signals. The quality of thresholds was visually assessed across a random subset of images to ensure sufficient signal selection and background rejection.

### 2.5 Image Processing

TPEF image intensities for both autofluorescence and IHC signals were normalized by laser power and PMT gain. These parameters were calibrated to fluorescein solutions of different concentrations, as described in detail elsewhere.^40^^,^^41^ Briefly, multiple solutions with known concentrations of fluorescein (μM range) were imaged for a range of PMT gain and laser power settings to establish a relationship between these parameters and image intensity. Laser power readings were acquired on each imaging day to account for daily variations. These measurements were then used to normalize the image intensities on each day and calibrate to the fluorescein concentrations. Whole-section images were stitched using Fiji’s (ImageJ) stitching plugin to compute the overlap between adjacent tiles, with a regression threshold of 0.05 and linear interpolation between the overlapping portions.^42^ After normalization and stitching, a mask was created to remove pixels with a low signal based on a manually identified threshold to mitigate noise from these regions. This mask was added to the manually drawn mask (see Sec. 2.5.1) and included in all subsequent analyses. The ORR was calculated for each pixel within all images using MATLAB (R2022a; MathWorks, Natick, Massachusetts). All histograms (including phasor plots^43^) were generated in MATLAB and scaled to the percentage of total pixels in the histogram. Specifics for processing different image types are explained in subsequent sections and graphically in Fig. 2.

#### 2.5.1 Masking and registration

Necrotic regions, non-tumor regions, and background were manually identified and masked out using a manual tracing program developed in MATLAB and converted to polygons using fast mapping.^44^ Matched sections with both autofluorescence and IHC image data were manually registered using a program developed in MATLAB. Briefly, two pairs of corresponding points are identified in each image. These points are used to define a line within the image. Necessary rigid transformations to align both lines are calculated. The line is first scaled, then rotated, and then translated. The final transform is rounded to account for the discrete pixels of images. The final transform is applied to the extracted IHC signal and mask from the IHC image. The registered IHC signals are stacked with the corresponding ORR map resulting in an M×N×3 image with ORR, pimo+, and HIF-1α+ layers. The intersection of the registered masks is used to create a new mask for the stack.

#### 2.5.2 Regional analysis of ORR, hypoxia, and HIF-1α accumulation

The registered stack (as described in Sec. 2.5.1) is used to perform a regional analysis of ORR, oxygenation, and HIF-1α accumulation within a 100  μm radius around each pixel. This radius represents the generally accepted maximum diffusion barrier of oxygen.^45^^–^^48^ This radius also mitigates the impact of minor registration errors introduced through the manual registration process, tissue movement during IHC staining, and variations in imaging depth between assays (differences in depth are limited by a section thickness of 50  μm).

The intersection of masks (as described in Sec. 2.5.1) was applied to each stack. The M×N×3 stack (of ORR, pimo+, HIF-1α+) was convolved with a 100  μm radius disk filter. The disk was created using MATLAB’s fspecial, which creates a circular averaging pillbox of a given radius (which may have fractional values on the border, see Fig. 2 for a visual representation). This filter was scaled up by the area of a circle to convert to a summation filter. Convolving results in a blurred M×N×3 stack with each 1×1×3 voxel representing the weighted sum of the signal within the disk region for the respective channel. The mask for each stack was separately convolved with the same filter to obtain a map of region size (in pixel counts) around each pixel. The blurred stack was divided element-wise by the map of region size to obtain the average of each signal for the region centered at all locations. This process is described graphically in Fig. 2.

#### 2.5.3 NAD(P)H lifetime analysis

Phasor plots of lifetime decays are, in short, a bivariate histogram of the real and imaginary parts of the Fourier transformation of the fluorescent decay for each pixel.^43^ An adapted MATLAB code was used to perform phasor transformations, based on work from Gottlieb et al.^49^ Phasor histograms for each group were manually inspected to ensure that they were reasonably approximated by two lifetime species. Under this assumption, the real and imaginary components of the transform (G and S coordinates, respectively) were fit using a simple linear regression in MATLAB. The intersection of this line with the universal circle was used to determine the short and long (free and bound, respectively) lifetime species ($\tau_{1}$ and $\tau_{2}$). All phasor points were then projected onto the fit-line, and the distance from the intersection points was used to calculate the free and bound intensity fractions ($\alpha_{1}$ and $\alpha_{2}$). The mean lifetime ($\tau_{m}$) was then calculated as the weighted average of short and long lifetime species for each pixel using the equation $\tau_{m}=\alpha_{1}\tau_{1}+\alpha_{2}\tau_{2}$.

#### 2.5.4 Multivariate histograms

All multivariate histograms were generated in MATLAB. Uni- and bivariate histogram counts were calculated using built-in histogram functions and normalized to the percentage of the total number of pixels within the group. Histogram counts were plotted against the center value of the bin. Bivariate histograms were plotted as a surface color-coded by the pixel percentage, and the shading between bin centers was interpolated linearly. Trivariate histograms were calculated by averaging the third variable (ORR in this case) within each bin’s range. The value of this average was used to update the color-code of the surface of the respective bivariate histogram. The pixel percentages were represented in the z-value of the surface plot.

### 2.6 Statistical Analysis

**Fig. 3:**
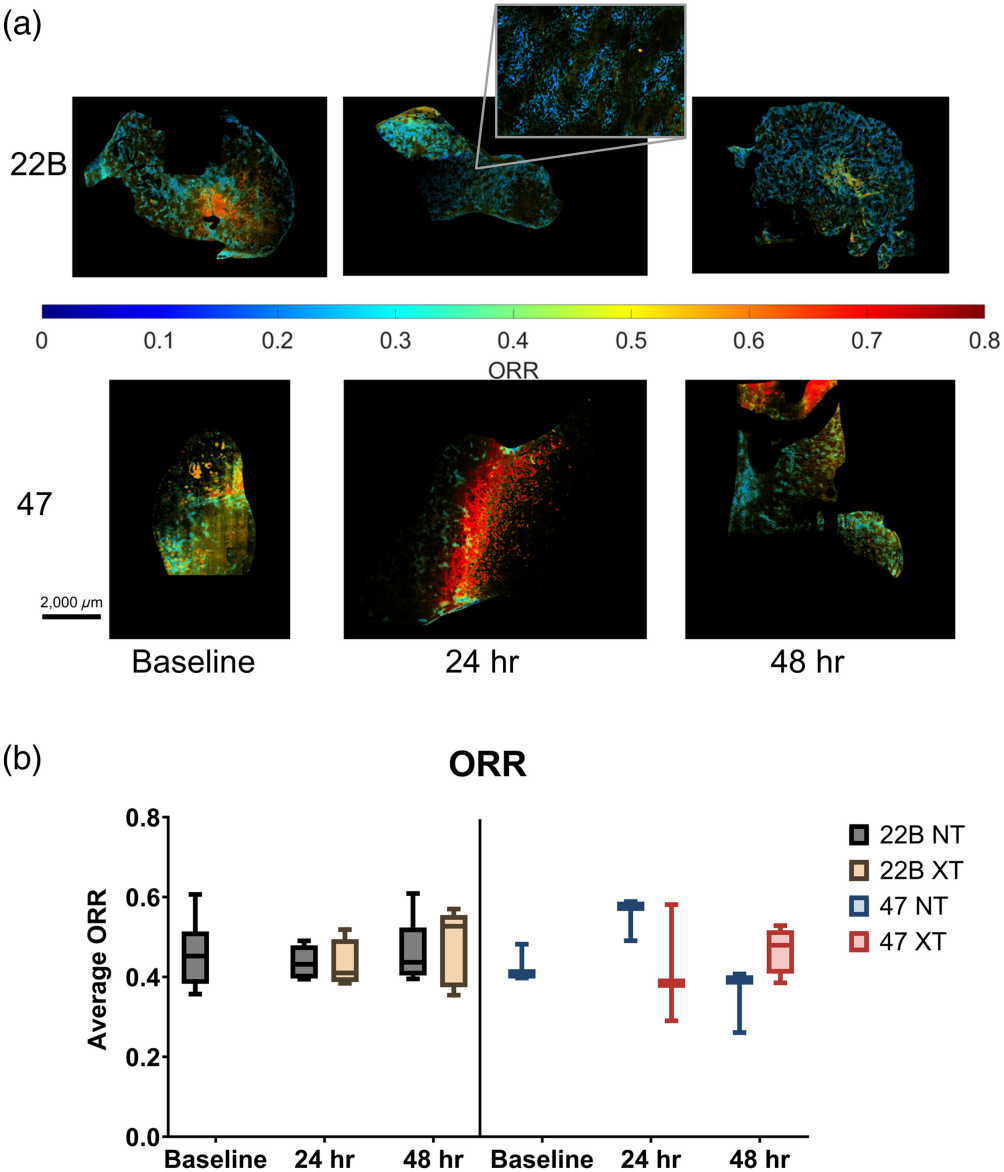
Whole-section image ORR (a) representative ORR maps of baseline and XT groups for each cell line with zoomed inset to show detail and (b) box-and-whisker plot of all samples in each group. $\mathrm{ORR}=\frac{I_{\mathrm{FAD}}}{I_{\mathrm{NAD}(P)H}+I_{\mathrm{FAD}}}$.

**Fig. 4:**
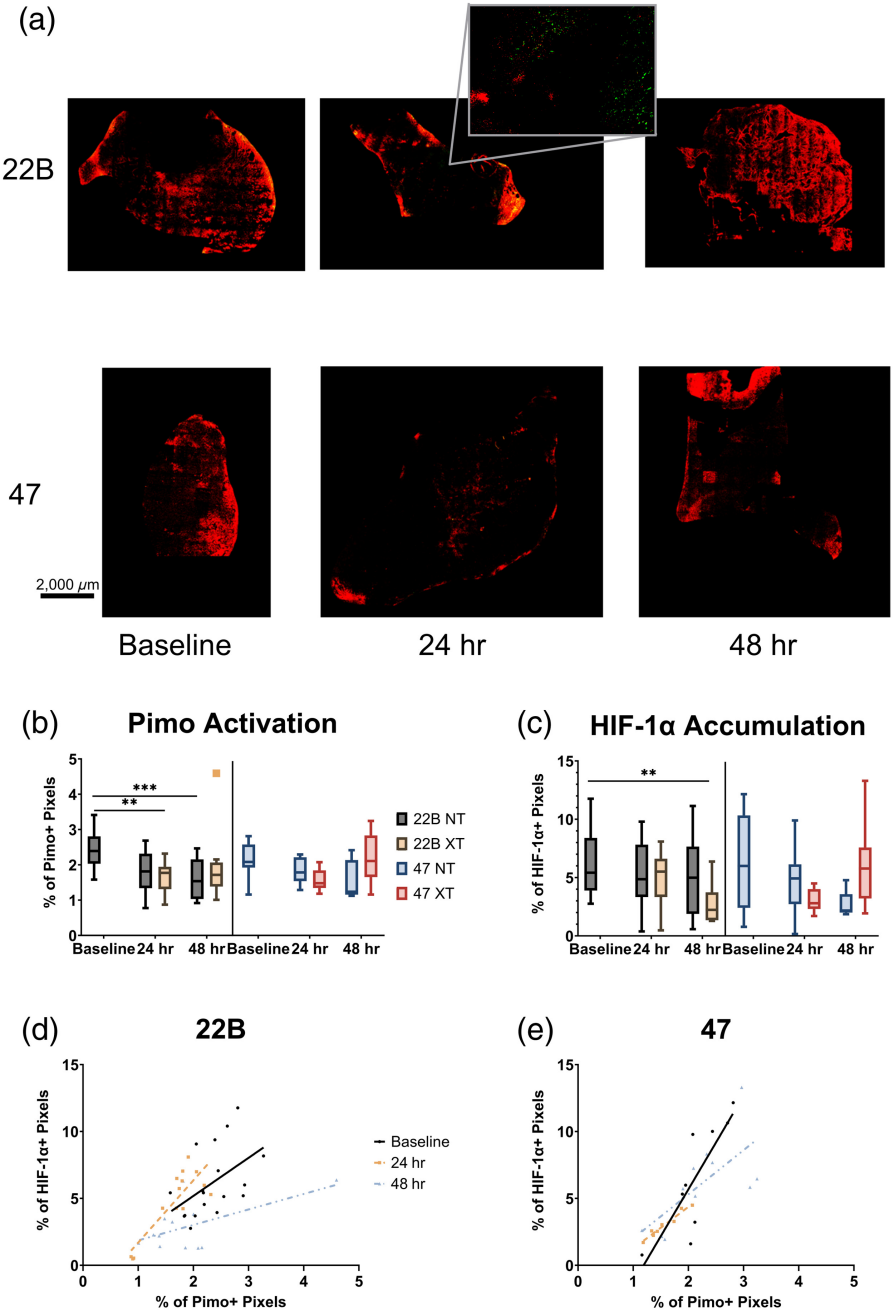
Whole-section image IHC (a) pseudo-colored representative images of baseline and XT groups for each cell line (pimo+ in red, HIF-1α+ in green). Zoomed inset (matched to the inset in Fig. 3) shows the punctate nature of HIF-1α distribution that contributes to the difficulty in visualization at large scales. Scale bar=2000  μm. (b) Box-and-whisker plot of pimo+ staining percent and (c) HIF-1α+ staining percent for all samples in each group (**: p<0.025, ***: p<0.005). (d), (e) Scatter plot with simple linear regression fit line superimposed for baseline and XT groups for pimo+ staining percent versus HIF-1α+ staining percent for (d) 22B and (e) 47 tumors.

For testing of bulk means for whole-section and ROI images [Figs. 3(b), 4(b), 4(c), 8(b), 8(d), and 8(f)], each sample and animal were initially tested with a multi-level nested ANOVA through a custom MATLAB script to ensure no significant variation due to the individual image or section. No significant variation was found due to these factors within any animal, so a simplified repeat-measures ANOVA was employed in JMP (SAS Institute, Cary, North Carolina) with means for each image treated as spatial repeats within the animal. A two-factor ANOVA for each cell line was used to analyze the variation due to treatment and time after radiation. Baseline metrics for both cell lines were compared using Student’s t-test (JMP). Post-hoc analyses were performed using Tukey’s HSD in JMP. Accordingly, all box-and-whisker plots are shown using Tukey’s method and generated in GraphPad Prism (Dotmatics, Boston, Massachusetts). Differences between interaction effects (treatment×timepoint) were not considered except with the baseline, and thus only pairwise differences between baseline, groups from the same treatment, or treatment groups with matched timepoint are shown and discussed.

Simple linear regressions [Figs. 4(d), 4(e), Figs. 9(b)–9(d)] were performed and plotted in GraphPad Prism (with the exception of phasor lines used to calculate mean lifetime and bound fraction as described in NAD(P)H lifetime analysis). Slopes of regression lines of individual datasets were compared using an ANCOVA (GraphPad Prism). Slopes and p-values for Pimo versus HIF-1α regression lines are included in Table 1 as m and $p_{\text{line}}$, and pair-wise ANCOVA p-values are listed (as relevant) with no subscript in the text. Slopes of phasor lines are included in figure legends [Figs. 9(b)–9(d)].

**Table 1:** Summary of correlation results for Pimo+% versus HIF-1α+% regressions. Slopes marked with ** are significantly different from one another (p=0.006).

Correlations between Pimo+% and HIF-1α+%						
	22B			47		
Timepoint	m	$R^{2}$	$p_{\mathrm{line}}$	m	$R^{2}$	$p_{\text{line}}$
Baseline	2.848	0.2401	0.0390	7.001	0.6735	0.0067
24 hr	4.652**	0.7292	0.0001	3.118	0.9524	<0.0001
48 hr	1.157**	0.3363	0.0481	3.301	0.4786	0.0127

**Fig. 5:**
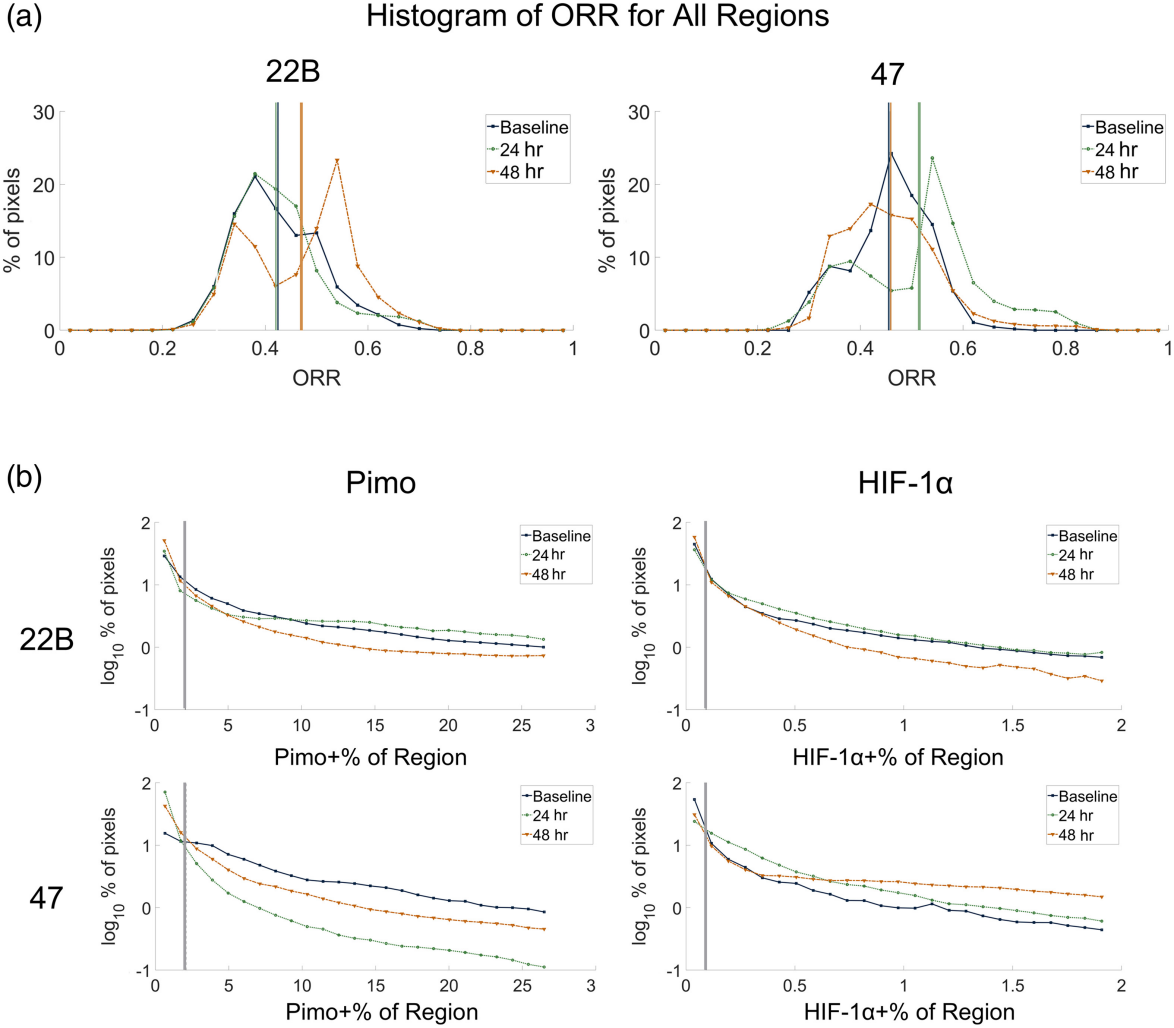
Univariate histograms of whole-section images for (a) ORR for baseline and XT groups for each tumor type with mean values for each group denoted by color-matched vertical lines, emphasizing the difference in information provided by bulk statistics compared with histogram analysis. (b) Histogram of the center quartiles of the regional percentage of pimo+ pixels and the regional percentage of HIF-1α+ pixels. Due to the skewed nature of the data, medians of all values for pimo+ and HIF-1α+ are denoted by the vertical dashed line, which was used as the cutoff for low/high pimo/HIF-1α. $\mathrm{ORR}=\frac{I_{\mathrm{FAD}}}{I_{\mathrm{NAD}(P)H}+I_{\mathrm{FAD}}}$

Univariate histograms were compared pairwise with a $\chi^{2}$ test for homogeneity on histograms bins (JMP) [Figs. 5, 7, and 10(b)]. Only pairs of interest were tested to limit the accumulation of type I errors. All reported p-values are adjusted (where relevant) using the Holm–Bonferonni method for multiple comparisons and employed manually. α=0.05 was considered statistically significant.

## 3 Results and Discussion

### 3.1 Analysis of Whole-Section Images of Hypoxia, HIF-1α, and ORR Shows Large Intra-Group Variances Within 22B and 47 Tumors

We determined the ORR for whole-section images at baseline and at each time point for the untreated controls and the treated groups (Fig. 3). For comparisons, only untreated controls (NT) versus treated (XT) groups at the same time point, baseline versus 24/48  h within each cell line, and baselines between cell lines were considered; we found no significant differences in the bulk ORR across these comparisons. However, we noticed large intra-group variances of the average ORR of tumor sections from both cell lines, suggesting the possibility of more complex distributions of ORR data that could obscure differences when considered in bulk.

For the whole-section IHC images (Fig. 4), we found that hypoxia was significantly lower in the treated 22B tumors at 24 h (1.655±0.465%) compared with baseline (2.401±0.497%; p=0.0108). However, there were no differences between the NT and XT groups at either 24 h or 48 h, suggesting that the observed decrease in hypoxia is likely not a result of treatment. We also found that hypoxia was significantly lower in the control 22B tumors at 48 h (1.889±0.922%) when compared with baseline (2.401±0.497%; p=0.0043) [Fig. 4(b)]. In fact, all groups are lower than the baseline in 22B tumors. We did not see any significant differences in hypoxic fraction in the resistant 47 tumors, though hypoxic fraction does appear elevated in the treated tumors at 48 h relative to the control tumors at the same time [Fig. 4(b)].

At 48 h in the treated 22B tumors (2.887±1.839%), HIF-1α accumulation was significantly decreased from baseline (6.161±2.588%; p=0.0148) [Fig. 4(c)]. Although not significant, there was also a notable difference between the NT and XT groups at 48 hr in the 22B tumor, with the XT tumors showing lower HIF-1α accumulation. No significant differences in HIF-1α accumulation were found in the 47 tumors, but the 48 hr XT group had elevated HIF-1α relative to the 48 hr control. This parallels the trend seen in hypoxic fraction at 48 hr between the NT and XT groups.

#### 3.1.1 Hypoxia and HIF-1α are positively correlated but with different slopes in the resistant and sensitive tumors

To investigate the relationship between hypoxia and HIF-1α, we used a simple linear regression of all whole-section image means for each time point [Figs. 4(d) and 4(e)]. All correlations were found to be significant and positively sloped (Table 1), confirming the expected correlation between hypoxia and HIF-1α. Although not reaching statistical significance, the slope of correlations at baseline is notably greater in the 47 tumors compared with the 22B tumors. In 22B tumors, this slope differs significantly between 24 and 48 hr [p=0.006; Fig. 4(d)]. No significant difference in slopes was found between time points in the 47 tumors (p=0.9425 for 24 hr versu.48 hr). With the exception of the treated 22B tumors at 24 hr, the slope of the correlation between hypoxia and HIF-1α is consistently greater in the 47 tumors, indicating a large HIF-1α accumulation for a small change in hypoxia and pointing to the possibility of non-hypoxic sources of HIF-1α accumulation.

#### 3.1.2 Distributions of ORR and HIF-1α A are significantly different at 24 and 48 hr following radiation compared with baseline in the resistant tumors

Having identified large intra-group variances in the analysis of bulk ORR, hypoxic fraction, and HIF-1α, we suspected that there may be sub-populations of metabolic phenotypes that were obscured when averaged over the entire tumor section. Therefore, we employed a histogram-based analysis of the entire section to further investigate the relationship between these three parameters (Fig. 5).

First, we compared all treatment groups with their respective baseline control for all regions. Only 47 tumors at 24 hr were found to be significantly different from their baseline control (p=0.0153), showing a peak above 0.5, whereas baseline and 48 hr groups have declining numbers of regions at this ORR [Fig. 5(a)]. The 22B tumors at 48 hr show a similar distribution of ORRs to that observed in the 47 tumors at 24 hr, though it was not statistically significant [Fig. 5(a)]. Next, we analyzed the distributions of regions for hypoxia and HIF-1α [Fig. 5(b)]. We found that the regional percentage of pimo+ pixels at 24 hr was significantly different from both baseline (p=0.0003) and 48 hr (p=0.012) in the 47 tumors, with a greater percentage of low-hypoxia regions and fewer high-hypoxia regions [Fig. 5(b) and Fig. S1 in the Supplementary Material]. The decrease in high-hypoxia regions at 24 hr is likely due to radiation-induced reoxygenation that we have observed in the 47 tumors^33^ and is concordant with the redistribution of the ORR toward higher values at 24 hr as seen here. By 48 hr, we observe an increase in high-hypoxia regions and a decrease in the ORR in the 47 tumors, which would be consistent with the development of a glycolytic phenotype under hypoxic conditions.^50^

#### 3.1.3 Low-pimo+, high-HIF-1α+ regions have populations with elevated ORR

**Fig. 6:**
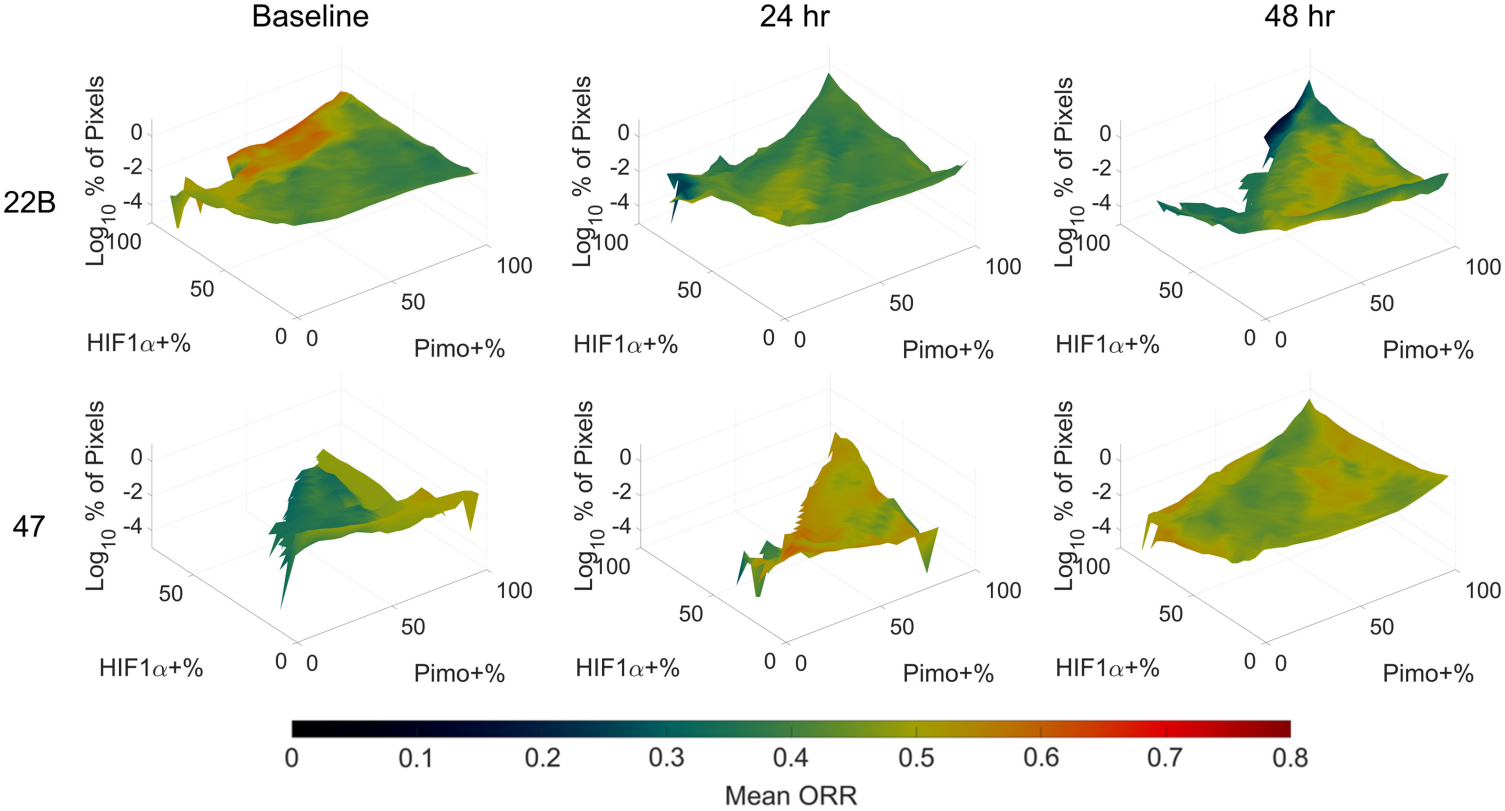
Trivariate histograms of $\log_{10}$ percent of pixels binned by the regional percent of pimo+ and HIF-1α+. The color bar corresponds to the mean ORR for each bin for all whole-section images. $\mathrm{ORR}=\frac{I_{\mathrm{FAD}}}{I_{\mathrm{NAD}(P)H}+I_{\mathrm{FAD}}}$.

We next performed a trivariate histogram analysis on these three factors (hypoxia, HIF-1α accumulation, and ORR) to determine potential distinct regions of sub-populations (Fig. 6).

The elevation of these histogram surfaces corresponds to the number of pixels within each bivariate bin of hypoxia and HIF-1α accumulation, spanning all possible combinations for all regions in the group. The color of the surface is the average ORR for the pixels that fall into each bivariate bin. Because of the skewed nature of the distribution of the regional percentage of both pimo+ pixels and HIF-1α pixels toward 0%, we used the median value of all regions (denoted as vertical lines in the figure) as a cutoff for low percentage and high percentage [Fig. 5(b)]. All regions with pimo or HIF-1α accumulation below the respective median, values are classified as low-pimo or low-HIF-1α regions. Similarly, all regions with pimo or HIF-1α accumulation above the respective median values are classified as high-pimo or high-HIF-1α regions. Qualitatively, the first point of note is the decrease in the number of regions with a low percentage of pimo+ pixels and high percentage of HIF-1α+ pixels (toward the top-left of xy-plane) in the 22B tumors at 48 hr (Fig. 6 and Fig. S2 in the Supplementary Material). In the 47 tumors, the opposite trend emerges, with an emergence of a small population of regions with a low percentage of pimo+ pixels and a high percentage of HIF-1α+ pixels at 24 hr and a much larger population at 48 hr. As discussed in Sec. 1, HIF-1α stabilization can be driven by a number of factors not limited to hypoxia. Here, we observe that regions with low-pimo, high-HIF-1α seem to be coincident with higher ORR sub-populations, particularly at baseline in the 22B tumors and in the 47 tumors at 24 and 48 hr post-radiation. In previous studies in cells *in vitro*,^32^ we have shown that an increase in ROS coincides with an increase in the ORR. Given that radiation-induced ROS can stabilize HIF-1α,^17^ these results appear to indicate the development of regions with non-hypoxia driven HIF-1α and an increase in ROS.

**Fig. 7:**
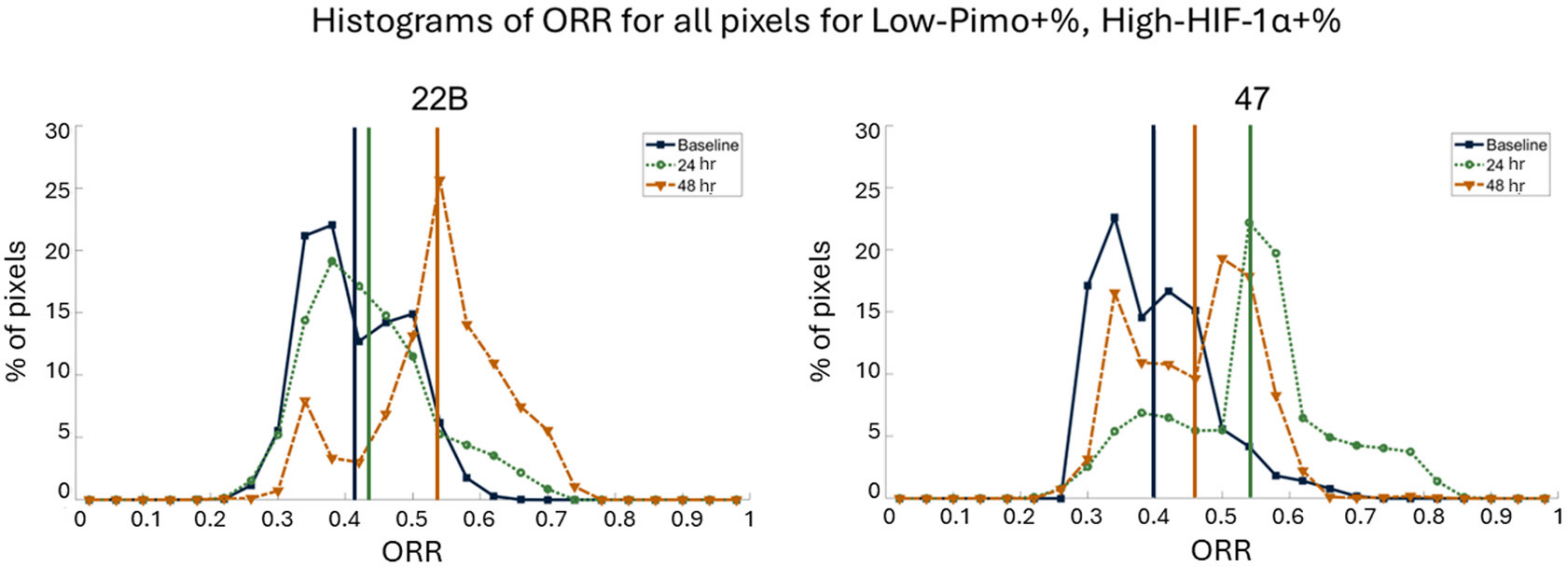
Histograms for ORR pixels in regions with a low percentage of pimo+ pixels and high percentage of HIF-1α+ pixels for both tumor types, also including means of the ORR values shown for each group in color-matched vertical lines. $\mathrm{ORR}=\frac{I_{\mathrm{FAD}}}{I_{\mathrm{NAD}(P)H}+I_{\mathrm{FAD}}}$.

Having observed these qualitative trends, we wanted to quantify differences in this specific region of the histograms. To do so, we used median values for all regional pimo+ and HIF-1α+ percentages from all tumors as the threshold for low/high regions [the median values are displayed as a dotted vertical line in Fig. 5(b)]. Using these values as criteria for consideration, we performed contingency table analyses on the distribution of the average ORR of regions that met the criteria (Fig. 7).

The distribution regional average ORR of low-pimo+, high-HIF-1α+ regions of 22B tumors at 48 hr was significantly higher compared with baseline (p≪0.0001), confirming the qualitative difference that we observed in the trivariate histograms. In the 47 tumors, the baseline distribution of the regional average ORR was different for the bulk [Fig. 5(a)] compared with the low-pimo+, high-HIF-1α+ regions (Fig. 7) (p=0.016). Further, the distribution of the average ORR of regions was significantly different at both 24 and 48 hr compared with baseline in the 47 tumors for low-pimo+, high-HIF-1α+ regions (p≪0.0001 and p=0.03, respectively). Distributions for high-pimo+ and high-HIF-1α+ regions are shown in Fig. S3 in the Supplementary Material.

### 3.2 ROI Analysis Reflects Trends Seen in Whole-Section Analysis

**Fig. 8:**
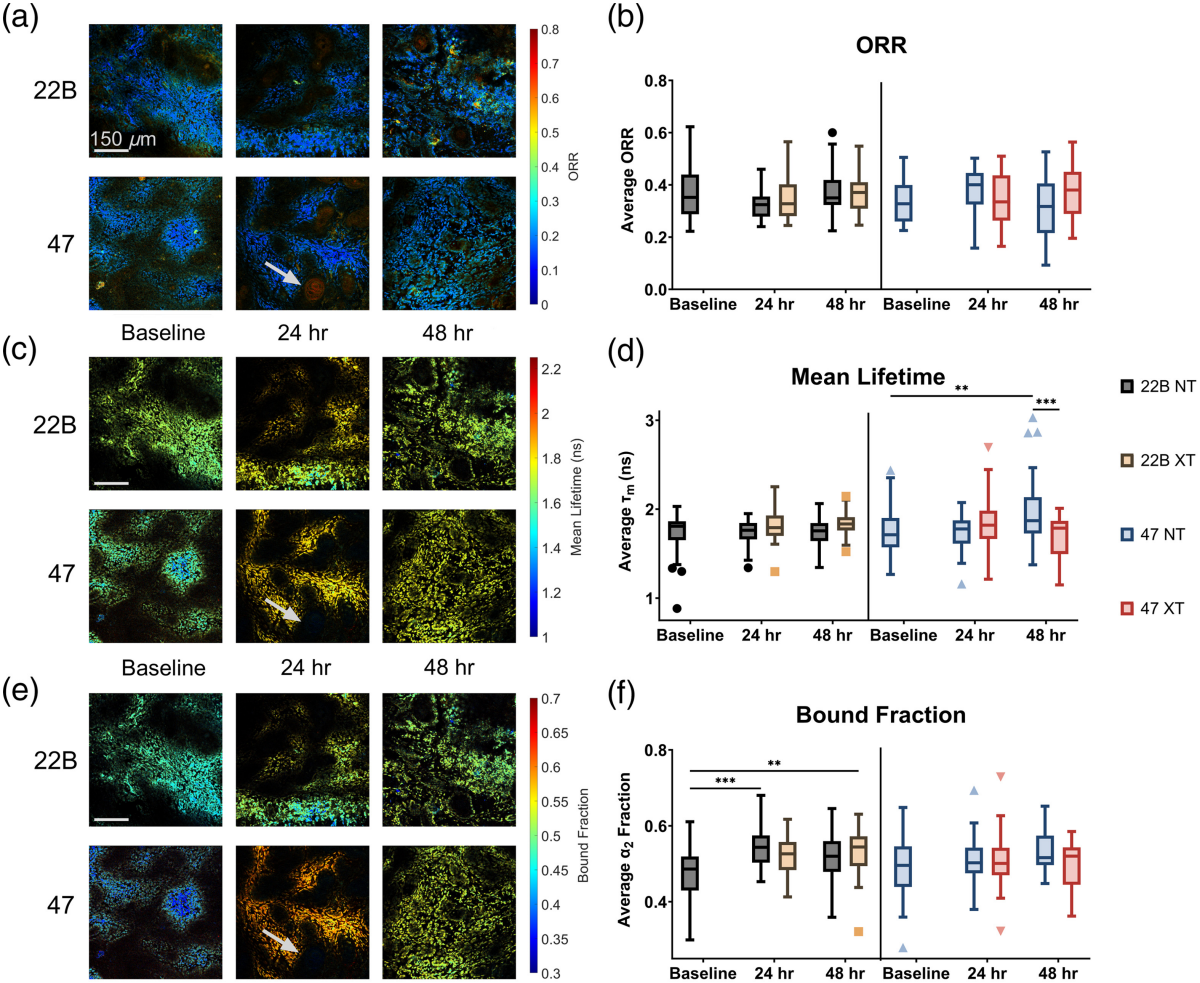
ROI imaging results for (a) and (b) representative ORR images and group ORR $\left(\mathrm{ORR}=\frac{I_{\mathrm{FAD}}}{I_{\mathrm{NAD}(P)H}+I_{\mathrm{FAD}}}\right)$, (c) and (d) mean lifetime ($\tau_{M}=\alpha_{1}\tau_{1}+\alpha_{2}\tau_{2}$), and (e) and (f) NAD(P)H protein-bound percent ($\alpha_{2}\%=\frac{\alpha_{2}}{\alpha_{1}+\alpha_{2}}$). (a), (c), (e) Color-coded representative ROI images for each output. Arrows point to an example of a mature keratin pearl as observed in each imaging modality. Images for each parameter are from the same ROI. Scale bar=150  μm. (b), (d), (f) Box-and-whisker plots of each parameter for all images within each group.

Figure 8 presents the representative images and group means from specific ROIs within the tumor section. The means of the parameters are calculated over the entire ROI shown here. Similar to the whole-section images, we saw no significant differences in the ORR for ROI groups [Fig. 8(b)].

All means were notably lower than the whole-section image counterparts. We did, however, observe similar relative trends in the bulk means for each group. We believe that the lower ORR is due to the selection of ROIs primarily while viewing 755 nm excitation, which could lead to a selection bias toward regions with marked NAD(P)H fluorescence. ROI images highlight the presence of distinct regions of keratinization [Fig. 8(a), see 47 tumors at 24 hr]. These are known as keratin pearls and are a marker of well-differentiated squamous cell carcinoma.^51^ The autofluorescent signal from these structures is dominated by FAD and keratin at 855 nm excitation, contributing a subset of regions with elevated ORRs relative to the bulk. Keratin has a broad emission spectrum that overlaps with both NAD(P)H and FAD and has been shown to contribute a significant 1.5 ns lifetime to FLIM measurements;^52^ however, excitation at 755 nm and emission in the blue channel mitigates much of the keratin interference, as observed [Figs. 8(c) and 8(e)]. In the 47 tumors, the 48 hr NT group was significantly elevated (1.988±0.405  ns) when compared with both the baseline (1.745±0.274  ns; p=0.0190) and 48 hr XT group (1.692±0.237  ns; p=0.0006). No significant differences emerged in the bound fraction of 47 tumors, though the relative trends tracked closely with those observed in the mean lifetime. In the 22B tumors, on the other hand, we found a significant increase in the bound fraction from baseline (0.481±0.072) of 24 hr NT tumors (0.544±0.047; p=0.0013) and 48 hr XT tumors (0.532±0.058; p=0.0077). This trend is reminiscent of the differences observed in the bulk hypoxic fraction. In that case, we saw a general decrease in hypoxic fraction over a 48 hr period relative to baseline, whereas here we see an increase in the bound fraction. These trends are consistent with previous work that has shown a decrease in the bound fraction with an increase in the hypoxic fraction.^50^

#### 3.2.1 Protein-bound NAD(P)H lifetime decreases in response to treatment in resistant tumors

**Fig. 9:**
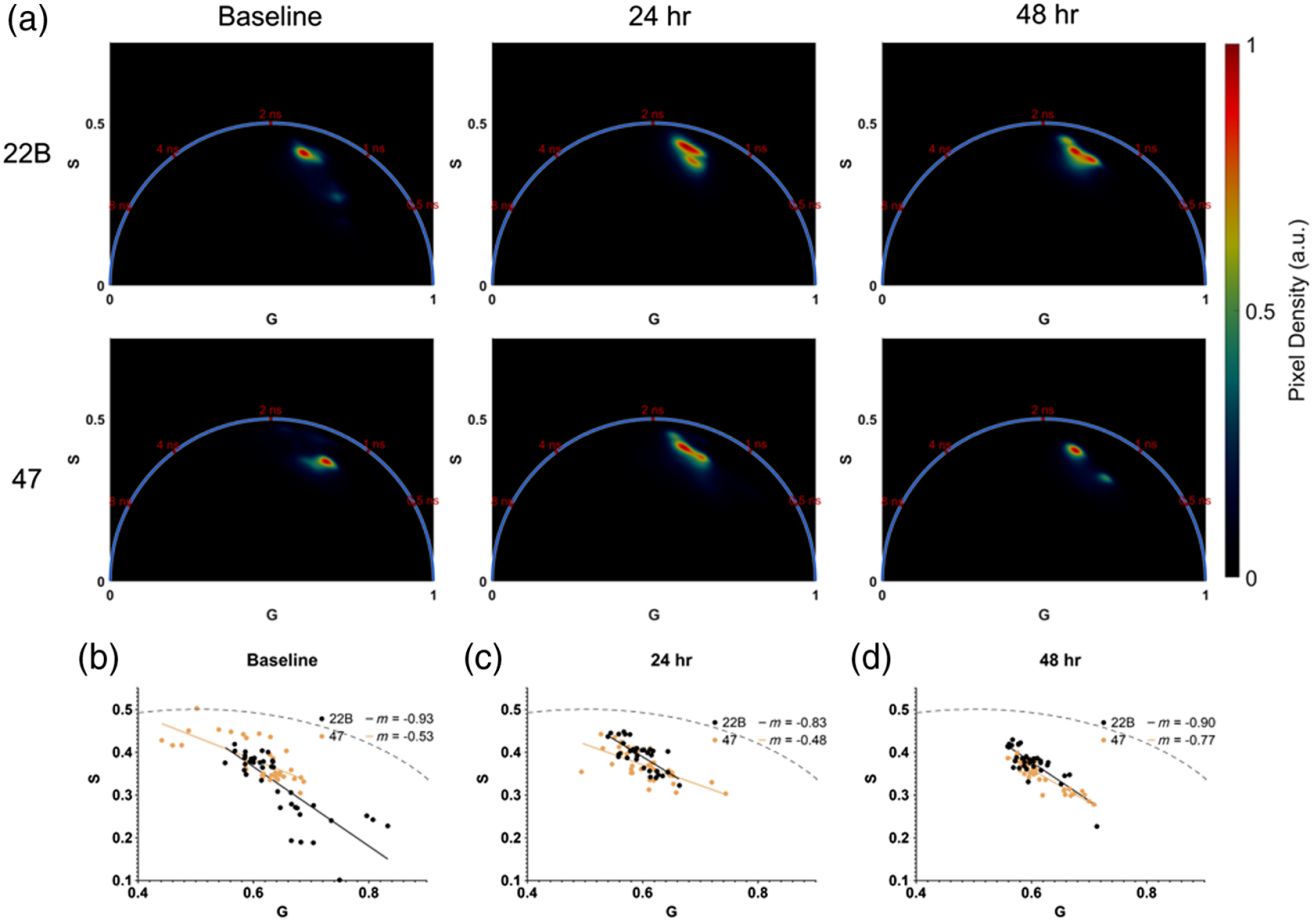
Bivariate histogram of phasor coordinates. (a) Phasor density plot after a single 3×3 median filter and threshold of 15 photons for 22B (top) and 47 (bottom) at baseline (left), 24 hr (center), and 48 hr (right). (b), (d) Scatter plots of average G and S coordinates for each ROI in the 22B and 47 tumors. For each plot, solid lines illustrate the linear regression fit, and the dotted line represents the universal circle. The slope of each fit is indicated in the figure legend.

Phasor plots (Fig. 9) support the presence of two primary NAD(P)H species for most pixels, evidenced by the elliptical nature of the phasor plot sub-populations oriented to two locations on the universal circle. Although there are multiple separate foci of pixels, due to the small number of animals in the study and the inability to follow animals longitudinally, we elected to analyze only whole-group phasors, rather than isolating sub-populations.

The means for each ROI were used to fit lines and compare groups [Figs. 9(b)–9(d)]. Although not reaching statistical significance, there is a noticeable difference between tumor types at baseline that diminishes by 48 hr. This is driven by a decreasing slope in the 47 tumors at 48 hr. A decreasing slope, in this case, accompanies a decrease in the lifetime of the long lifetime (protein-bound NAD(P)H) component as the intersection point on the universal circle rotates clockwise toward shorter lifetimes.

#### 3.2.2 High NAD(P)H bound fraction is associated with lower ORR at baseline in sensitive tumors and in response to treatment in resistant tumors

**Fig. 10:**
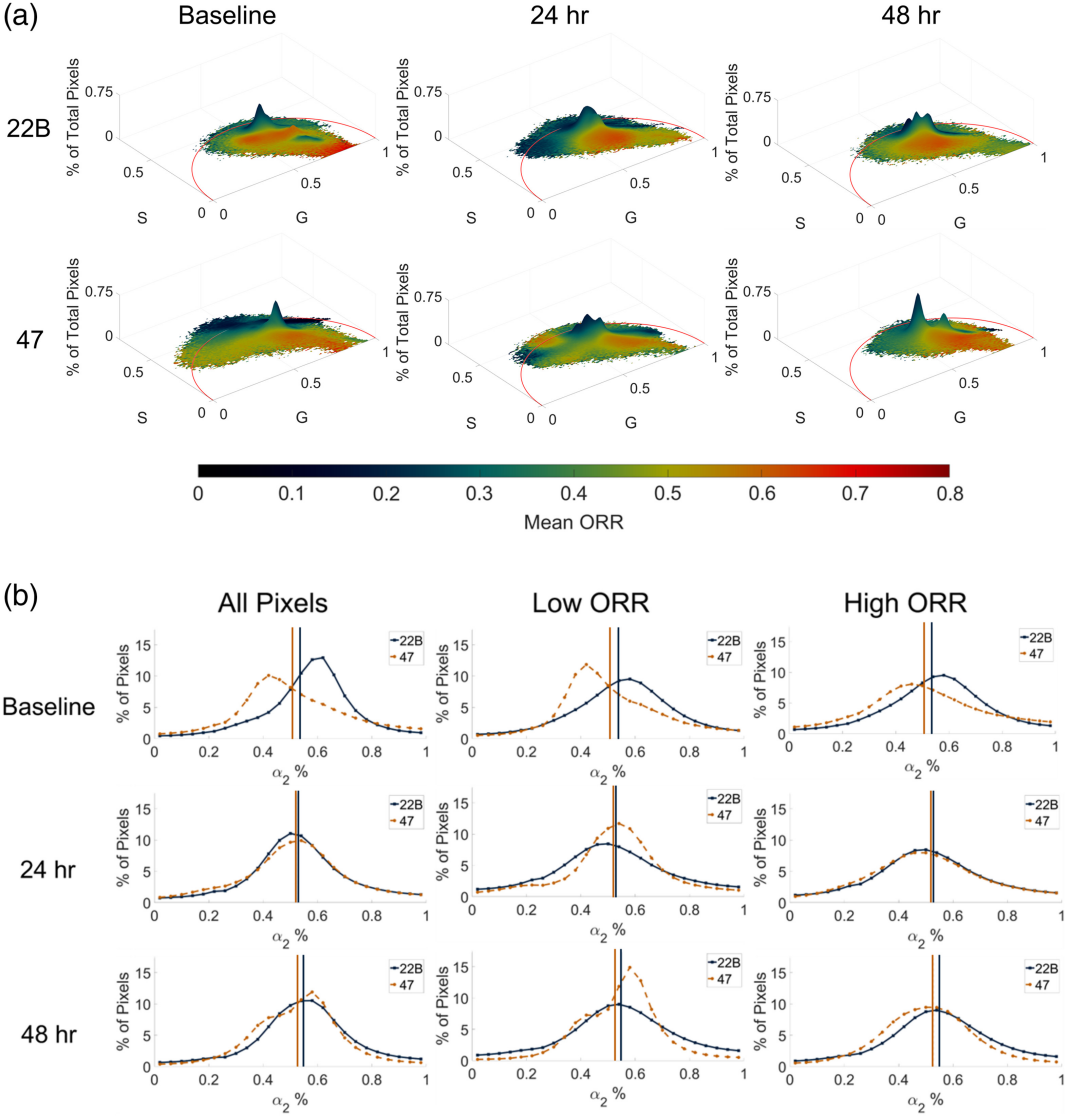
Multivariate histogram analysis of ROI images. (a) Trivariate histograms of phasor coordinates after a single 3×3 median filter and threshold of 15 photons. Color bar corresponds to the mean ORR for each bin for all ROIs in 22B (top) and 47 (bottom) at baseline (left), 24 hr (center), and 48 hr (right). (b) Histograms of bound fraction ($\alpha_{2}$) from phasors for all pixels (left), pixels with a low ORR (middle), and pixels with a high ORR (right) at baseline (top), 24 hr (center), and 48 hr (bottoms) for all ROIs of 22B and 47 tumors. Low and high cutoffs were determined using Otsu’s method for the distribution of the ORR for all groups. $\mathrm{ORR}=\frac{I_{\mathrm{FAD}}}{I_{\mathrm{NAD}(P)H}+I_{\mathrm{FAD}}}$; $\alpha_{2}\%=\frac{\alpha_{2}}{\alpha_{1}+\alpha_{2}}$.

To investigate how the subtle differences in lifetime may be associated with the difference in metabolism, trivariate histograms of the phasor coordinates and ORR were created and inspected [Fig. 10(a)].

Initially, it was evident that different ORR species tend to cluster together in phasor space. Noticeably, lower ORR species tend to be closer to the universal circle and have a higher bound fraction (which is equivalent to being near the long lifetime component or, in this study, near G=0.6 and S=0.4). Higher ORR species tend to be closer to the short lifetime component (toward G=1 and S=0) comparatively, but also shifted inward from the universal circle in all phasors. This suggests a greater mix of short- and long-lifetimes and possibly more than two lifetime species. The exact location of both of these ORR species seems to depend on the tumor and time after treatment. Guided by the trivariate histogram, we investigated lifetime endpoints (bound fraction and mean lifetime) for low- and high-ORR species, using the mean of all ORRs within the analysis as the threshold value [Fig. 10(b) and Fig. S4 in the Supplementary Material].

In general, the bound fraction and mean lifetime are highly correlated, so only the bound fraction is presented here. We manually determined that mean lifetime trends were similar. Matched histograms for mean lifetime are available in Fig. S4 in the Supplementary Material. Although no differences reach statistical significance in this analysis, there is a trend evident in both tumor types. The 22B tumors show a decrease in bound fraction after treatment, particularly in species with a low ORR. The 47 tumors have a steady mean across all times for all pixels, but the distribution for low-ORR species at 48 hr shows an increasing number of pixels with a higher bound fraction. Interestingly, this difference in the distribution is not evident in high-ORR regions. A high bound fraction with a low ORR has been associated with fatty acid synthesis in mesenchymal stem cells and mechanistically is consistent with these results, as glycolysis outpaces oxidative phosphorylation to provide precursors for fatty acids, and NADH is enzymatically utilized to synthesize fatty acids.^27^^,^^50^ *De novo* lipogenesis protects cancer cells from external insults, such as oxidative stress, and the inhibition of lipogenesis increases oxidative stress-induced cell death.^53^ Studies have identified increased levels of fatty acid synthase (FASN) in radiation-resistant head and neck cancer cells.^54^^,^^55^ FASN is a key player in lipogenesis and has been shown to be a prognostic indicator of radiation resistance in clinical nasopharyngeal carcinoma.^56^

## 4 Conclusion

Hypoxia and glucose metabolism play key roles in determining the efficacy of radiation therapy. In this study, we used high-resolution imaging of endogenous fluorescence to evaluate the response to a single dose of radiation treatment in sensitive and resistant tumor xenografts across multiple dimensions: ORR, NAD(P)H lifetime, and IHC of hypoxic fraction and HIF-1α accumulation. By acquiring high-resolution images at multiple time points (before, 24 hr after, and 48 hr after treatment), we sought to understand the spatiotemporal relationship between hypoxia, HIF-1α, and metabolism. To our knowledge, the relationship between these three parameters has not been examined in this manner. We present a data processing and visualization approach to identify patterns in the relationship between these three parameters and provide a framework for future studies to investigate similar multivariate relationships. Multivariate histograms plot the frequency of pixels within a range for each of the variables. Although uni- and bivariate histograms are commonplace, we propose a method for effectively visualizing and analyzing trivariate histogram data. Histograms such as this can effectively preserve the spatial information regarding co-localization of variable quantities. Our analysis reveals relationships and distinct sub-populations within the tumor microenvironment that did not necessarily follow bulk trends. Although this study included a relatively small number of animals in each group, the methods and initial results from such analyses presented here, we believe, hold promise for understanding the complex energy economy of the tumor microenvironment. Understanding the complex relationship between tumor oxygenation and metabolism will contribute to the development of therapies that can overcome treatment resistance in the clinic.

Table 2 summarizes the key observations of this study. In the radiation-sensitive 22B and, to a greater extent, the radiation-resistant 47 tumors, we observed an increase in the ORR associated with regions of low-hypoxia and high-HIF-1α at 24 and 48 hr after radiation therapy.

**Table 2:** Summary of key multivariate observations from all analyses. Each row summarizes trends that were observed together through histogram analyses.

Summary of observations						
Tumor type	Hypoxia	HIF-1α	ORR	$\alpha_{2}$	Possible driver	Figures
22B, 47	↓	↑	↑	—	ROS-activated HIF-1α^32^	Figs. 6 and 7
22B, 47	↑	↑	↓	—	Hypoxia-activated HIF-1α	Fig. 6, Fig. S3 in the Supplementary Material
47	—	—	↓	↑	Fatty-acid synthesis^50^	Fig. 10

As discussed earlier, although these regions are likely associated with an increase in radiation-induced ROS based on previous work *in vitro*, we do not have data corresponding to ROS labeling here that can confirm this observation. We found reduced ORRs in regions of high-hypoxia and high-HIF-1α both within the 22B and 47 tumors. This observation is consistent with an expected increase in glucose catabolism in hypoxic regions that leads to a buildup of NADH within the mitochondria. We also observed an increased ORR, albeit to a lesser extent, in the 47 tumors at 24 hr in regions of high-hypoxia and high-HIF-1α (Fig. S3 in the Supplementary Material). Although we do not fully understand the reason for this increase, it could be attributed to the generation of reduced glutathione; the oxidation of NADPH to ${\mathrm{NADP}}^{+}$ in a reaction catalyzed by glutathione reductase generates reduced glutathione, which can then scavenge free radicals. Depending on the contribution of NADPH to the overall NAD(P)H autofluorescence, the oxidation of NADPH to ${\mathrm{NADP}}^{+}$ can lead to an increase in the ORR. However, we were unable to confirm these trends due to a lack of complementary lifetime data from whole-section images. FLIM was only performed on ROIs and not whole-section images due to the total time needed to generate adequate photon counts. IHC signals confounding endogenous signals precluded ROI analysis with hypoxic fraction and HIF-1α accumulation. The ability to co-register images of hypoxic fraction, HIF-1α, and FLIM would have also provided stronger evidence to support fatty acid synthesis as a possible reason for the observation of an increase in the bound fraction of NAD(P)H along with a decrease in the bulk hypoxic fraction and HIF-1α in the sensitive 22B tumors. In addition, the use of IHC in this study necessitates imaging of *ex vivo* sections that are either frozen or formalin fixed. Freezing and thawing for metabolic imaging has been reported in previous studies to affect ORR measurements and metabolite levels, namely, increasing the measured ORR relative to fresh tissue controls.^38^^,^^41^^,^^57^ Importantly, these same studies also observed consistent trends within frozen groups. Taken together, this supports the analysis of data when comparing between groups that were fixed consistently, as in this study, but also highlights the need for *in vivo* studies of these relationships as a necessary step to a clinically applicable understanding of radiation resistance.

Even within whole-section images, our analysis is limited by registration quality. Pixel-level registration would be ideal to maximize the resolution of multivariate relationships. Such a high-quality resolution, however, is precluded by IHC staining, which requires imaging to be performed on different days, making it nearly impossible to ensure identical imaging depths. Furthermore, the process of IHC itself is likely to cause tissue sections to move, tear, or fold, which further inhibits pixel-level registration. Therefore, we performed our image analysis on whole-section images by considering 100  μm regions around each pixel. We chose to use 100  μm to approximate the diffusion limit of oxygen from capillaries.^45^^–^^48^ Convolving large image stacks is computationally intensive, and we chose to use only a single disk size to limit computational necessities. Future studies investigating how these relationships may change with region sizes could be beneficial particularly because hypoxia and hypoxia-stabilized HIF-1α are known to vary spatially and temporally and are known to accumulate at different distances from vasculature.^58^

Although it is standard to present either lifetime curve fit data or phasor plots, we chose to include both (Figs. 9 and 10 and Figs. S4 and S5 in the Supplementary Material) to illustrate the benefits of this multivariate histogram approach for multiple data types. Phasor analysis and curve-fitting methods each have pros and cons.^59^^,^^60^ Lifetime-fitting introduces assumptions and simplifications that may not be valid—namely, a two-species fit—resulting in a long-lifetime parameter that is not directly analogous to any single species, but rather a weighted average of bound species’ lifetimes.^27^^,^^61^ These fits, however, provide easy-to-interpret outputs that can be quickly compared. Phasor analysis, on the other hand, does not require any *a priori* assumptions regarding the number of lifetime species to be included in the analysis, but it can be difficult to quantify and compare across groups.^43^^,^^59^ The use of a linear fit to phasor coordinates reintroduces assumptions, but these assumptions can be validated before they are implemented. Ultimately, phasor analysis may provide a more robust tool for metabolic analysis when combined with complementary endpoints, such as the ORR.

## Supplementary Material

10.1117/1.BIOS.1.1.015003.s01

## Data Availability

The data supporting the results presented in this article are available on GitHub (https://github.com/jiversivers/umscc_data).
