# Erratum: Investigating the relationship between hypoxia, hypoxia-inducible factor 1, and the optical redox ratio in response to radiation therapy

**DOI:** 10.1117/1.BIOS.1.3.039801

**Published:** 2024-12-16

**Authors:** Jesse D. Ivers, Nagavenkatasai Puvvada, Charles M. Quick, Narasimhan Rajaram

**Affiliations:** a University of Arkansas, Department of Biomedical Engineering, Fayetteville, Arkansas, United States; b University of Arkansas for Medical Sciences, Department of Pathology, Little Rock, Arkansas, United States

## Abstract

The article clarifies a correction to the published article: Column 1 in the third row of Table 2 was corrected to be 47 rather than 22B.

The original article was published in Volume 1 Issue 1 of *Biophotonics Discovery* (BIOS) on 28 May 2024 with an error in Table 2. Column 1 in the third row of Table 2 should be 47 rather than 22B.

Upon correction, the article was republished under the same DOI (10.1117/1.BIOS.1.1.015003) on 12 December 2024.

