# Supplementary material for: Investigating the relationship between hypoxia, hypoxia-inducible factor 1, and the optical redox ratio in response to radiation therapy
Source: Biophotonics Discov. 2024 May 28;1(1):015003. doi: 10.1117/1.BIOS.1.1.015003 (PMC11922545; doi:10.1117/1.BIOS.1.1.015003)
Supplement: Supplementary file 1 [file BIOS_001_015003_SD001.pdf]

Investigating the relationship between hypoxia, hypoxia-inducible factor (HIF-1), and the optical redox ratio in response to radiation therapy

Supplementary Figures

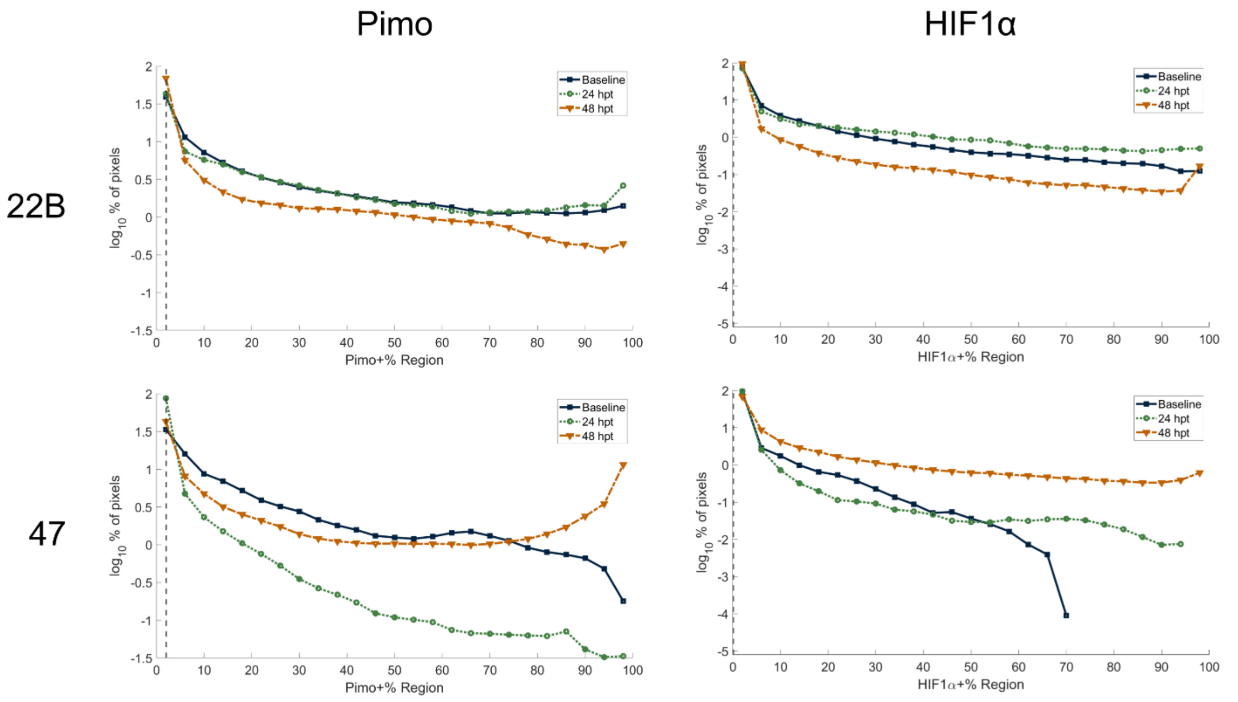

Supplementary Figure 1: Histograms of regional percentage of Pimo+ (left) and HIF-1α+ (right) pixels for all regions ORR 22B (top) and 47 (bottom) tumors with medians for each group shown as dashed vertical line. The median line for the HIF-1α plot is very close to the y-axis.

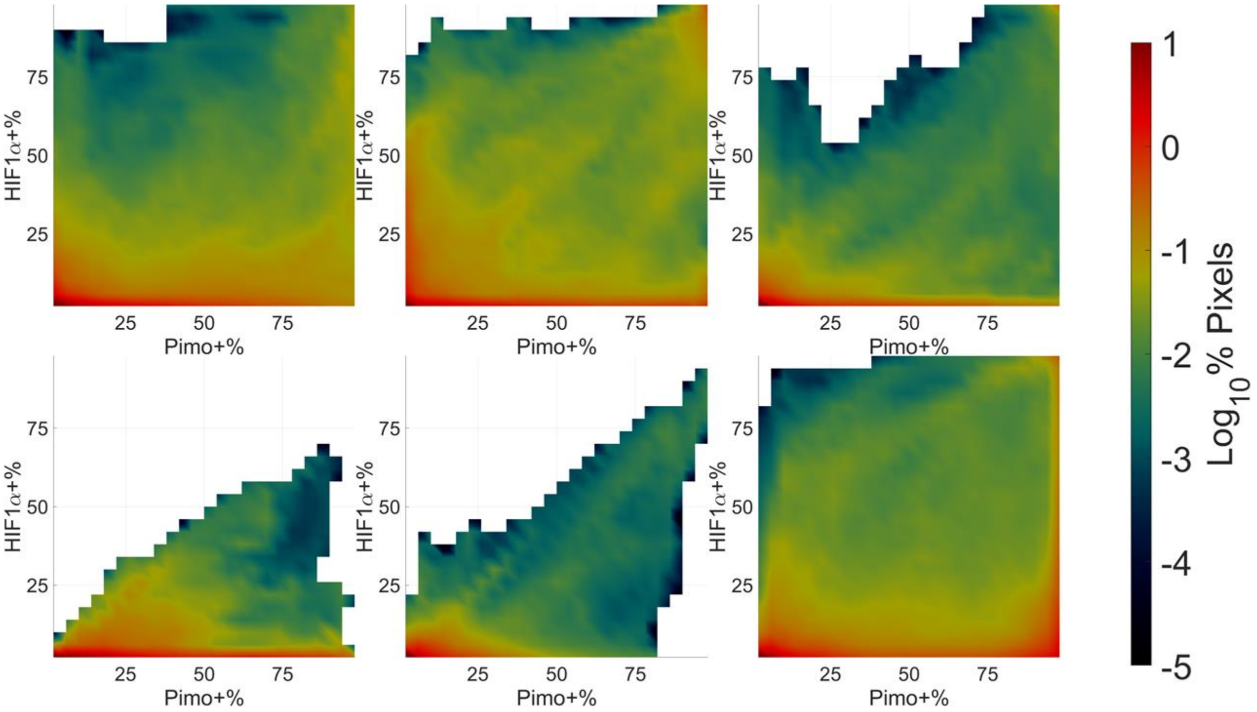

Supplementary Figure 2: Bivariate histogram of regional fractions of pimo and HIF-1α for all whole-section images in 22B (top) and 47 (bottom) at baseline (left), 24 hr (center), and 48 hr (right).

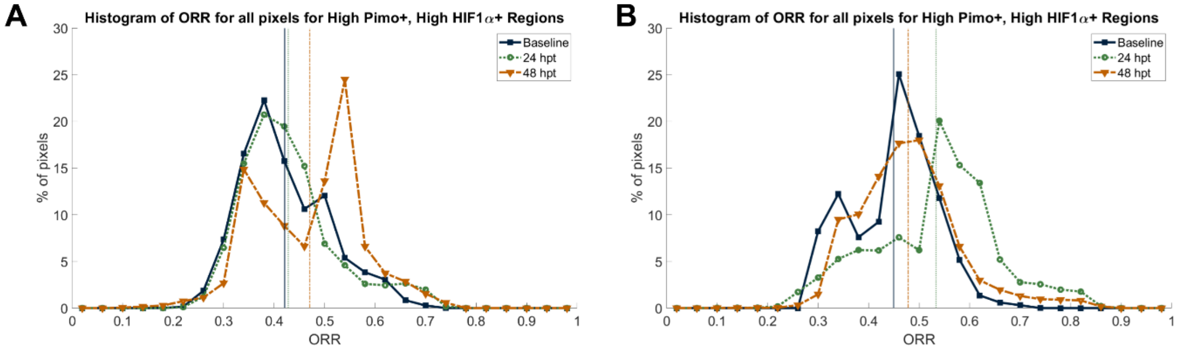

Supplementary Figure 3: Histograms of average ORR of high-Pimo+, high-HIF1α+ regions in (A) 22B and (B) 47 tumors with means for each group shown as vertical color-matched lines.

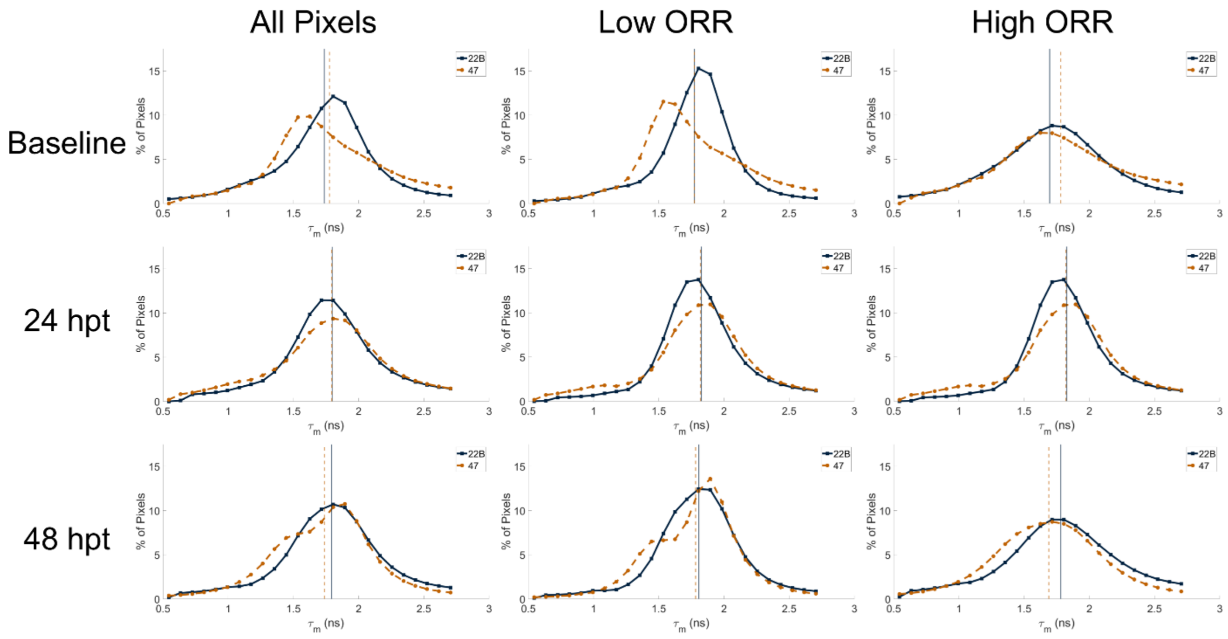

Supplementary Figure 4: Histograms of mean lifetime (τ_M) in ns from phasors for all pixels (left), pixels with low-ORR (middle), and pixels with high-ORR (right) at baseline (top), 24 hr (center), and 48 hr (bottoms) for all ROI of 22B and 47 tumors. Low and high cutoffs were determined using Otsu's method for the distribution of ORR for all groups.

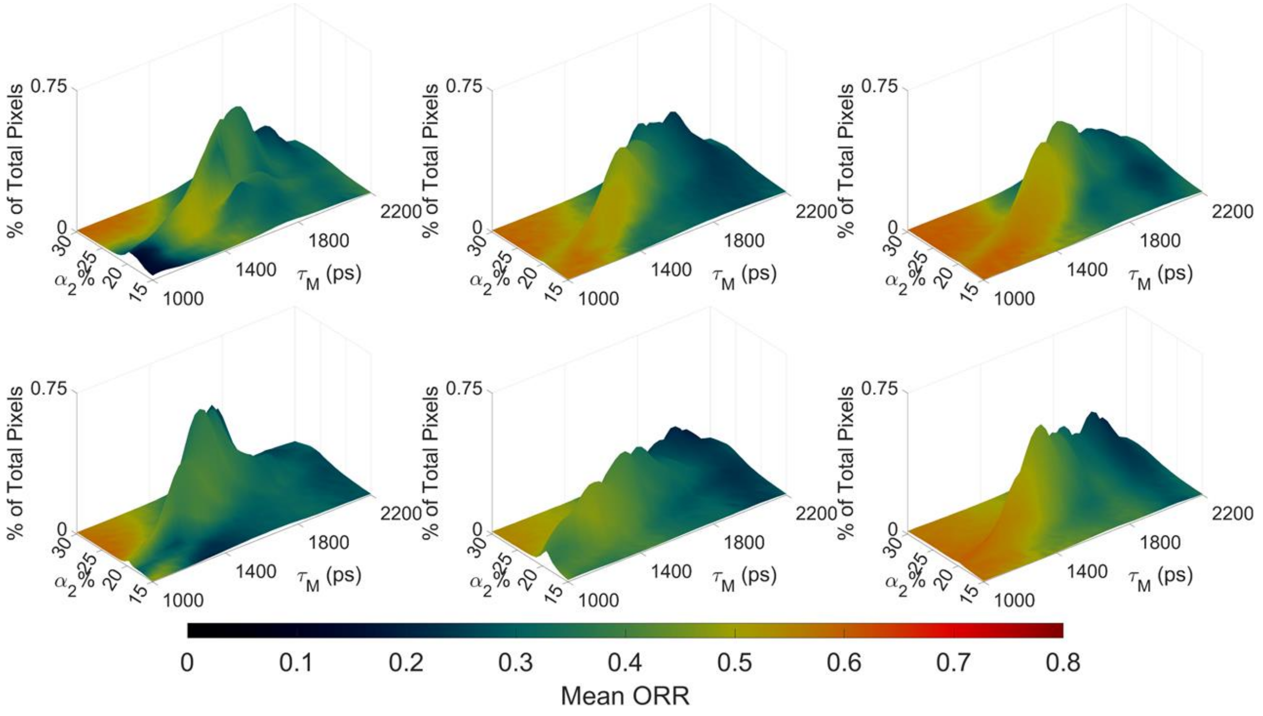

Supplementary Figure 5: Captures of trivariate histogram of fit parameters τ_M and α_2% colored by mean ORR for each bin for all ROIs in 22B (top) and 47 (bottom) at baseline (left), 24 hr (center), and 48 hr (right).
